# Retinal pigment epithelium atrophy after epiretinal membrane and internal limiting membrane peeling: case reports

**DOI:** 10.22336/rjo.2022.16

**Published:** 2022

**Authors:** Nicolás Rivera-Valdivia, Hiroshi Maeda-Yasunaga, Pablo Cabal-López, Carlos Salgado-Cerrate, Carlos Abdala-Caballero

**Affiliations:** * Grupo Oftalmológico Abdala-Figuerola AF, Barranquilla, Colombia

**Keywords:** retinal pigment epithelium atrophy, epiretinal membrane, internal limiting membrane, pars plana vitrectomy, dyes

## Abstract

**Objective:** To describe the development of retinal pigment epithelium (RPE) atrophy after uncomplicated pars plana vitrectomy (PPV) with epiretinal membrane (ERM) and/or internal limiting membrane (ILM) peeling in 2 patients.

**Cases description**:

Case 1: A 79-years-old female with diagnosis of a full-thickness macular hole in her right eye (OD) with best corrected visual acuity (BCVA) of: 20/100 and left eye (OS): 20/70. After surgery she developed large RPE hyperplasia and presented hand movement that did not improve with pinhole.

Case 2: A 69-years-old female patient who had ERM in her OS with BCVA of 20/30 in both eyes (OU). PPV was assisted with brilliant blue (BB) to better visualize the ILM. During follow-up visits we evidenced RPE atrophy in the zone where peeling was done. In the last control after 2-years, her visual acuity was 20/40 that did not improve with pinhole.

**Discussion:** There are three possible mechanisms to explain this complication: toxic damage, mechanical trauma during the membrane removal with forceps, or a combination of both. In our cases, a combination of them is probably the cause of the presence of RPE atrophy.

**Conclusion:** Vitrectomy with membrane removal is successful in most cases with low rate of complications. Because RPE atrophy is infrequent, our suggestion is to continue performing this technique and if possible, it should be done without dye staining to minimize risks.

**Abbreviations:** ERM = epiretinal membrane, ILM = internal limiting membrane, MH = macular hole, RPE = Retinal pigment epithelium, OD = right eye, BCVA = Best corrected visual acuity, OS = left eye, OU = both eyes, IOL = intraocular lens, OCT = Optical coherence tomography, BB = Brilliant blue, TB = Trypan blue, ICG = indocyanine green

## Introduction

Epiretinal membrane (ERM) is a fibrovascular tissue that forms over the internal limiting membrane (ILM) [**1**,**2**]. It can be idiopathic or secondary to retinal vascular diseases, trauma, ocular tumors, uveitis, intraocular surgical procedures, retinal tears, among others [**2**,**3**]. The ILM is the basal lamina of the neurosensory retina formed by Müller cells [**4**] and is closely related to the formation of macular holes (MH). The management of ERM can be to observe if there is no compromise of visual acuity nor metamorphopsia, or to surgically remove in case of symptoms [**1**,**3**,**5**]. However, the handling of idiopathic MH tends to be surgical in most cases [**6**,**7**]. The procedure consists of pars plana vitrectomy (PPV) with ERM removal associated or not with ILM peeling in the first case; and ILM peeling in the second case [**4**,**7**]. Some complications are cataract progression, ocular hypertension, retinal tear or detachment, vitreous hemorrhage, macular edema, ERM recurrences and more rarely retinal pigment epithelium (RPE) atrophy, among others [**3**,**8**].

We present the case of 2 patients who developed post-surgical RPE atrophy; the first one underwent ILM peeling for MH and the second one surgery for ERM associated with ILM removal. Both surgeries were performed by an expert retina surgeon, without any complications. 

To the best of our knowledge, these are the first case reports that describe RPE atrophy following PPV in South America.

## Case description

**Case 1**

79-year-old female, with no ophthalmic history, was referred by general ophthalmologist for the management of a MH and cataract in the right eye (OD). She was evaluated by a retina specialist in our center confirming the diagnosis. 

**Fig. 1:**
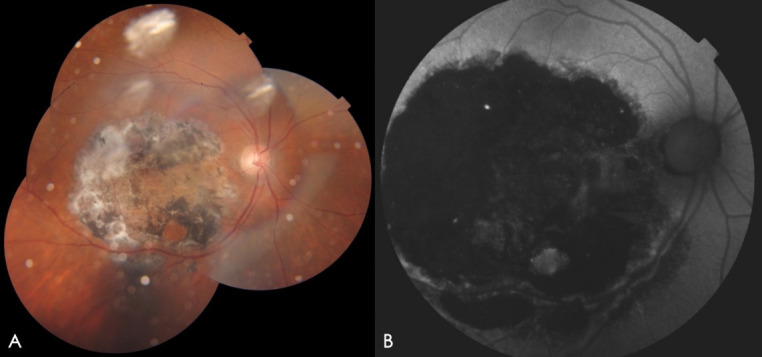
Images of OD. **A.** Fundus color photography shows circular RPE atrophy area in the zone of MLI peeling. **B.** Central hypoautofluorescence that correlates with image **1A**

**Fig. 2:**
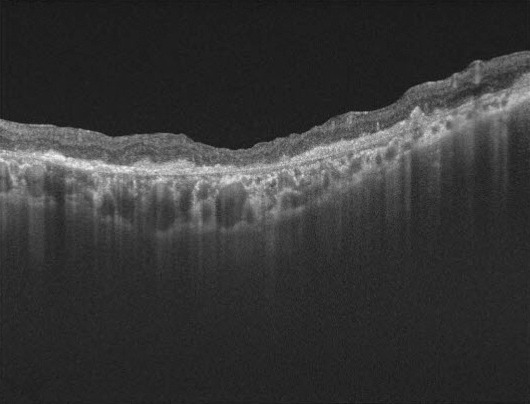
OCT of OD reveals enlargement of RPE associated to an altered inner/outer segment junction

Best corrected visual acuity (BCVA): OD 20/100 and left eye (OS) 20/70. At slit lamp, anterior segment was unremarkable and NO4 cataract in both eyes (OU) was found. Fundus examination showed OD with MH larger than 600 microns and adjacent mild RPE atrophy; OS healthy macula with applied retina. It was decided to perform combined surgery of PPV + ILM peeling + gas tamponade + phacoemulsification with intraocular lens (IOL) implant in OD. No dyes were used and the procedure was performed without complications. Subsequently, routine controls were made during the first month. BCVA OD at 1-month was counting fingers at 2-feet, which did not improve with pinhole. Fundus examination showed a closed MH with severe hyperplasia of the central macular RPE. At 2-months follow-up, OD fundus color photography evidenced large RPE atrophic changes in the peeling zone (**Fig. 1A**), marked hypoautofluorescence at the same level (**Fig. 1B**), and macular optical coherence tomography (OCT) showed loss of retinal layers parallelism, irregular RPE enlargement with disruption at inner/outer segment junction and posterior optical reinforcement (**Fig. 2**). After 2-years, BCVA was hand motion in the OD with persisting rounded hyperplasia of the RPE corresponding to the size of the peeling zone.

**Case 2**

69-year-old female, with no ophthalmic history, came to our center because of metamorphopsia in her OS and a diagnosis of ERM and bilateral cataract given by a general ophthalmologist. She was evaluated by a retina specialist in our center confirming the diagnosis. 

BCVA was 20/30 OU. At slit lamp, anterior segment was unremarkable and P2 cataract in OU was present. Fundus exam showed ERM with applied retina OU. It was decided to perform a combined surgery of PPV + ERM peeling + gas tamponade + phacoemulsification with IOL implantation in OS. During the procedure, ERM was removed without any dye and ILM removal was stained with brilliant blue (BB). Peeling of ILM was difficult because of a great adherence of the membrane to the retina, therefore the procedure was performed without any further complications. Routine controls were scheduled during the first month. At 2-months follow-up, macular edema with OS central macular thickness of 327 μ was observed in OCT, so an Ozurdex® was implanted decreasing this value to 262 μ. After 5-months of follow-up, marked atrophy of the macular RPE with adjacent pigmentary changes was observed.

**Fig. 3:**
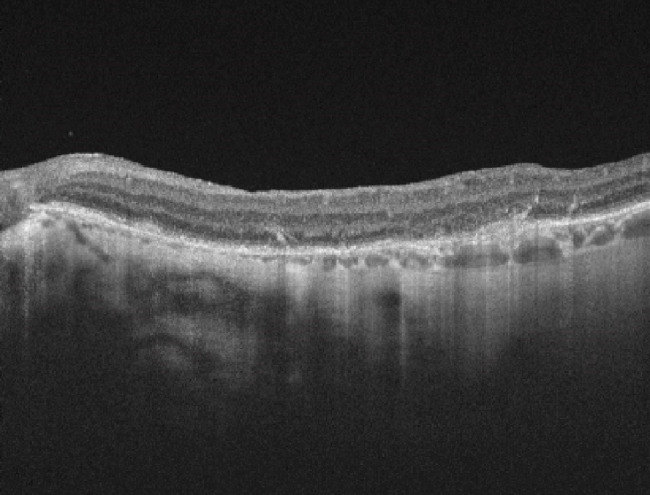
OCT of OS presents loss of retinal layers parallelism and irregular RPE with ellipsoid zone disruption

**Fig. 4:**
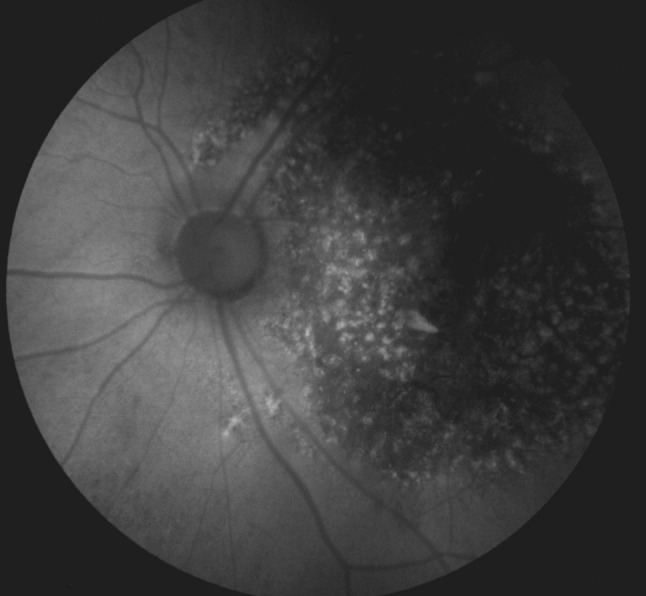
Fundus autofluorescence of OS shows RPE mottling pattern with macular compromise

A new macular OCT of the OS was requested, showing loss of parallelism of the retinal layers, irregular RPE and disruption of the inner and outer retina compatible with atrophy at that level (**Fig. 3**); fundus autofluorescence showed an extensive RPE mottling characterized by an hypoautofluorescent area in the posterior pole extending outside the temporal vascular arcades associated with hyperreflective dots (**Fig. 4**).

In the last control 2-years after surgery, BCVA was 20/40 in the OS that did not improve with pinhole.

## Discussion

PPV with membrane peeling is the procedure of choice to treat MH and ERM, especially when they decrease visual acuity and produce metamorphopsia secondary to macular anatomy disruption [**2**,**5**]. In the case of ERM, it can be associated with ILM peeling to decrease the recurrence rate, improve macular anatomy and enhance visual outcomes [**1**,**5**]. However, as shown in a review by Diaz-Valverde et al., there are surgeons against this technique, who argue that when recurrence occurs, the visual compromise is not significant and it can cause iatrogenic damage on Müller cells [**5**]. It is important to consider that sometimes the peeling of both membranes occurs at the same time due to the adherence they present [**1**].

This technique can be assisted by dyes, such as BB, trypan blue (TB), dual blue or indocyanine green (ICG), which serve to stain membranes [**1**,**2**,**4**,**8**,**9**]. Chromovitrectomy is a useful tool to facilitate membranes visualization, reduce operating time and minimize mechanical trauma [**8**]. However, some publications suggest toxic damage in retinal cells [**4**,**5**,**10**,**11**]. 

In our first case, we did not use any dyes to peel the ILM because of a good visualization of the membrane, and in the second case, ERM was removed without staining and then we used BB to perform the ILM peeling.

Several authors have reported different complications, some more frequent than others [**3**]. Atrophy of the RPE has been described as infrequent in some series, often related to dye assistance peeling. Nevertheless, as reported by Donati et al. [**3**] and as in one of our cases, it may be present in patients in whom dyes were not used since membranes were accurately visualized without the need for staining. In 2021, Ortiz et al. [**12**] presented a case of RPE atrophy following PPV assisted by BB in a high myopic MH. Inoue et al. [**6**], Uamoto et al. [**7**] and Engelbrecht [**13**] reported some cases associated with ICG use. Jain et al. [**14**] and Saeed et al. [**15**] published 2 cases after vitrectomy assisted by TB. In literature, 3 possible mechanisms to explain these secondary alterations are described: direct toxicity from the stains, mechanical traction on the pigment epithelium during removal with forceps [**1**,**3**-**5**,**10**], or a combination of them. Mechanical damage is probably the reason in our cases, due to the important adherence between membranes and retina, chiefly in the second case. Also, intraoperative retinal phototoxicity from the microscope and the endoilluminator must be considered as potential cause of RPE atrophy [**12**].

## Conclusion

Membrane removal is indicated in all cases of MH and in the presence of ERM when metamorphopsia and/or decrease in visual acuity are present. These procedures are widely performed worldwide with excellent results in most cases, and as any surgery, they are not exempt from complications. However, these are infrequent and should not be a limitation to continue performing them. The peeling of ILM in ERM surgery remains controversial, nonetheless in our extensive experience, anatomical and functional outcomes are very good with a low rate of complications. For this reason, we believe that both membranes could be removed at the same time. Moreover, if membranes are visualized adequately, the suggestion is to avoid the use of dyes because of the risk of toxicity.

Larger studies with high methodological quality should be carried out to understand the pathophysiology of this complication in order to prevent it and improve final outcomes.

**Conflict of Interest**

The authors declare no conflict of interest.

**Informed Consent and Human and Animal Rights statements**

Informed consent has been obtained from all individuals included in this study.

**Authorization for the use of human subjects**

Ethical approval: The research related to human use complies with all the relevant national regulations, institutional policies, is in accordance with the tenets of the Helsinki Declaration, and has been approved by the Ethics Committee of Grupo Oftalmológico Abdala-Figuerola AF, Barranquilla, Colombia.

**Acknowledgements**

None.

**Sources of Funding**

None.

**Disclosures**

None of the authors has any financial interest to disclose.
